# Academic Effects of the Use of Flipped Learning in Physical Education

**DOI:** 10.3390/ijerph17010276

**Published:** 2019-12-31

**Authors:** Francisco Javier Hinojo Lucena, Jesús López Belmonte, Arturo Fuentes Cabrera, Juan Manuel Trujillo Torres, Santiago Pozo Sánchez

**Affiliations:** Department of Didactics and School Organization, University of Granada, 18071 Granada, Spain; fhinojo@ugr.es (F.J.H.L.); jesuslopez@ugr.es (J.L.B.); arturofuentes@ugr.es (A.F.C.); jttorres@ugr.es (J.M.T.T.)

**Keywords:** educational innovation, digital learning, experimentation, learning impact, didactic benefits, primary and secondary education

## Abstract

The technological characteristics of today’s society have favored the inclusion of information and communication technology (ICT) and the emergence of new training methodologies in educational spaces. This study addresses flipped learning as an innovative approach in the teaching and learning processes of physical education at two educational stages, primary and secondary education. The objective of this study is to analyze the effectiveness of flipped learning with respect to traditional methodology. A descriptive and correlational experimental research design was used through a quantitative perspective. Two study groups were established, one control (traditional methodology) and one experimental (flipped learning) in each educational stage. A total of 119 students from an educational center in Ceuta (Spain) participated. These participants were chosen intentionally. The data were collected through a questionnaire. The results show that the experimental group obtained better evaluations in the academic indicators, highlighting the motivation, autonomy, and interactions between the different agents. Regarding the effectiveness of flipped learning according to the educational stage, its potential was demonstrated in both stages, highlighting a significant improvement in autonomy in secondary education.

## 1. Introduction

In today’s society, there is wide use of information and communication technology (ICT), being part of the usual practice in people’s daily lives [1]. In particular, in the field of education, the inclusion of ICT has been reflected in current and innovative teaching processes [2], both in the action of teaching on the part of the teachers [3] and in the way students learn, highlighting the potential offered by educational technology [4].

All the great changes that have occurred in education in recent years have been caused by the use of technology in the service of the educational community [5]. This has encouraged improvements in educational actions, increasing the motivation and availability of a wide list of techno-pedagogical resources [6,7]. Thus, it also encourages better access to content for students [8], who welcome the use of ICT in their training process [9].

In this sense, it can be affirmed that ICT has become a fundamental means [10] for the teaching and learning processes that are currently being developed [11] and the creation of new spaces dedicated to training [12] and development of innovative learning experiences [13]. All this is oriented to the search for quality in education, typical of an era digitalized in all areas [14].

This inclusion of technology in the educational spectrum is necessary so that teaching methodologies can adapt to the times and the concerns of today’s students. One result is so-called flipped learning, a methodological approach created by Jonathan Bergmann and Aaron Sams. These experts, in 2012, developed online audiovisual materials with content that students had to learn, allowing all students to access the content and customize their learning at their own pace [15]. Currently, this training method has been gaining increasing popularity, being carried out in numerous classrooms at all educational levels, as it is very practical and effective in instructional processes [16,17,18]. The novelty of this work is to take the use of flipped learning to an area little explored in emerging methodologies, physical education.

The pedagogical basis of flipped learning focuses on the use of time when students are outside the classroom to interact with the content [19,20]. This is carried out through digital platforms and tools generated by teachers [21,22,23]. The investment of this approach lies in using the teaching period to develop didactic actions based on the previous experience of students, who interact with the content autonomously through digital channels [24,25,26]. In this way, greater motivation of students and greater interaction on the part of all participants in education are achieved, as reflected in previous studies [27,28].

Therefore, flipped learning is postulated as an effective training method, demonstrated by the number of studies carried out that record its benefits, such as the high rate of commitment demonstrated by students [29], and improved participation in [30] and motivation toward tasks [31]. In addition, it causes increased self-control and individual regulation of learning by students [32], who adapt and regulate learning at their own pace and according to their own needs [31,33]. This increases peer relationships and greater socialization of everyone involved in the education process [34,35]. It also leads to an improvement in the resolution of problems posed during learning [36,37].

All the benefits presented so far affect learning outcomes [38], showing an improvement in scores obtained by students on assessment tests [39], as well as in the acquisition of competencies and set objectives [40,41,42]. Therefore, the flipping of learning moments [43] also generates effective empowerment of overturned thinking and critical thinking in students [44,45,46]. All this causes a positive attitude by students toward learning [47].

In this sense, flipped learning can be considered as a techno-pedagogical approach with a high rate of effectiveness compared to other, more traditional methods [48,49,50]. In the classical methodologies, every informative channel circulates through the teacher as an indisputable figure of the teaching and learning process [51], disregarding the prominence and limiting the actions of students themselves.

All these reported benefits in the scientific literature can be extrapolated to all the knowledge that students must acquire, especially knowledge that requires a more practical type of action. In this line, physical education becomes an ideal subject in which to develop flipped learning [52]. Specifically, in recent research carried out on physical education students, the potential of this innovative training approach has been demonstrated [53,54,55].

Consequently, innovation in the subject of physical education is already a reality, as stated by studies of its specialized impact, where various innovative practices are carried out to achieve improvements in academic indicators [56,57,58,59].

### Justification and Objectives

Given the characteristics of the society in which students develop today, there is a need to innovate and continue implementing new ways of teaching and learning in different subjects of the official curriculum, one of which is physical education. Physical education is precisely a field of study that is currently being highly researched, considering it as a very important element for student development [60,61]

This subject assumes a relevant role due to the increased sedentary lifestyle as a consequence of changes in the daily habits of adolescents due to technology [62]. On this basis, technology should not be considered as negative and harmful to student health, but quite the opposite: we should take advantage of its potential attraction and motivational power [63,64] to awaken in students new attitudes toward physical activity.

It is for this reason that in this work the flipped learning methodological approach is used for the development of physical education sessions, with the purpose of fulfilling the need for education based on new challenges, means, and techno-pedagogical resources that contemporary society offers [65]. Following this line of educational innovation through flipped learning, it must be taken into consideration that its effectiveness will be closely conditioned on the characteristics of the students [66] and its applicability will vary according to the educational stage where it is implemented [67].

The purpose of this study is to follow the line of other recent research confirming the effectiveness of this model of pedagogical innovation [16,17,18,45,46,47,48,68] versus the more classical and conservative styles, which are still used in learning spaces [69]. Specifically, in this work the focus of study is the primary and secondary education stages, as the first stages where students are more in contact with the technology that surrounds them and they have access to [70].

Therefore, the objective of this research is to verify the effectiveness of flipped learning in physical education in the development of traditional training actions, from the perspective of two distinct educational stages, primary and secondary. The following specific objectives are based on this statement:

- To know the variability of the motivation, autonomy, critical thinking, resolution of problems, and use of class time of students according to the methodology used.
- To find out the degree of interaction of students with their peers, their teachers, and the content according to the methodological approach carried out.
- To determine the versatility of the ratings of evaluation tests based on the methodology used.

## 2. Materials and Methods

### 2.1. Research Design and Data Analysis

The study was approached from a quantitative point of view, through an experimental design of a descriptive and correlational nature, following the guidelines of the experts [71,72]. To this end, two group typologies were defined, control and experimental, at each educational stage, primary and secondary. In the control group, the teacher developed traditional training, while in the experimental group there was an innovative teaching and learning process based on flipped learning. This establishes that the teaching methodology used assumes the role of an independent variable and the efficacy achieved is raised as the research-dependent variable.

The analysis of the data was carried out using the statistical package for the social sciences (SPSS) version 24(International Business Machines Corporation, New York, NY, USA). Basic statistics such as mean (M) and standard deviation (SD), and other specific statistics such as Fisher’s skewness (Skew) and Pearson’s kurtosis (Kurt), used to determine the trend in the distribution of the data, are covered in this version. The comparison of means between the control and experimental groups was realized by means of the Student’s t-test. Cohen’s *d* and bi-serial correlation (*r*) were also used to measure the size of the effect. A statistically significant difference of *p* < 0.05 was established throughout the analysis.

### 2.2. Participants

Since this is an experimental study, it does not require a large volume of participation, as in other studies [73,74]. In this research, the sample was made up of 119 students from an educational center in the Autonomous City of Ceuta (Spain), the particularities of which are shown in Table 1. The focused and concrete context of the participants is justified in the search for differentiating findings with respect to other research carried out on flipped learning, as it is a region with certain characteristics at a social, geographical, multicultural, and inclusive level [75,76].

Particularly, the subjects of study were enrolled in the sixth year of primary education (*n* = 60, boys = 26, girls = 34, M_AGE_ = 12 years, SD = 1.01) and the fourth year of secondary education (*n* = 59, boys = 23, girls = 36; M_AGE_ = 16 years, SD = 1.26). The sampling technique for selecting participants was for convenience of a nonprobability nature, due to the ease of access to learners.

### 2.3. Instrument

An ad hoc questionnaire was used to collect the data, taking some validated instruments on the state of the matter [64,77,78]. The questionnaire consisted of 42 items. It was divided into two parts well differences. In the first block, different sociodemographic variables such as gender, age, city of residence, nationality, religion, academic year, repetition of course, learning difficulty, availability of technological resources, type of technological resources, and rating in evaluation tests were collected (only in post-test). In a second block two factors are collected, the attitudinal factor composed of five dimensions (motivation, autonomy, critical, resolution, class time) and an interactive factor composed of three dimensions (teacher, classmates, content).

The inclusion criteria of these dimensions and variables were (a) to formulate items that would allow the collection of social, educational, attitudinal, and interactive data about students; (b) to write items briefly and concisely; and (c) to consider the observations of the experts. The exclusion criteria were (a) to eliminate items that caused confusion or interpretation problems; (b) to avoid a large number of items in the questionnaire; and (c) to avoid similar answers that would cause doubt in the participants.

The items are presented mainly in a Likert response format, on a scale of four valuation points, with one the lowest and four the most positive score.

The instrument was validated both qualitatively and quantitatively. The former was carried out using a Delphi method, made up of 10 doctors from different Spanish universities. The inclusion criteria for selecting experts were (a) experience with and studies on educational technology; (b) specialist in the field of activity and physical education; (c) carrying out innovative practices in their professional development; and (d) in-depth knowledge of flipped learning.

These specialists gave a positive evaluation of the questionnaire (M = 4.98, SD = 0.41, min = 1, max = 6), and offered a series of recommendations to optimize the instrument, based on reducing the number of questions and improving the wording of some of them, with the aim of favoring interpretability and completion of the questionnaire. In turn, the feedback was analyzed by Fleiss’ kappa (k) and Kendall’s W statistics to determine the concordance and relevance of the judgments made (k = 0.86, W = 0.88). Quantitative cut validation was then carried out using exploratory factor analysis, following principal components analysis (PCA) with varimax rotation. Dependence between the variables was formulated by Bartlett’s test of sphericity (2643.52; *p* < 0.001) and relevant sample adequacy was found by the Kaiser–Meyer–Olkin test (KMO = 0.89).

In addition, the internal structure was analyzed by confirmatory factor analysis with the maximum likelihood technique, with statistically significant estimated parameters and those with factor loads greater than 0.56 achieving saturation of latent variables. Various adjustment indices that reached adequate values were used (χ^2^/df = 2.09, Goodness-of-fit statistic (GFI) = 0.98, Adjusted goodness-of-fit statistic (AGFI) = 0.97; Comparative fit index (CFI) = 0.96, Normed-fit index (NFI) = 0.97, Tucker-Lewis Index (TLI) = 0.98, Root mean square error of approximation (RMSEA) = 0.043), revealing a sustainable model.

Finally, the results obtained for each of the analyzed dimensions are presented, both of the attitudinal factor (Table 2) and the interactive one (Table 3). The results show good reliability indices, the total of the scale being an α value of 0.86, composite reliability of 0.84, and average variance extracted of 0.82. Similarly, no convergent validity problems Average Variance Extracted (AVE) > 0.5 or discriminant, Maximum shared squared variance (MSV) > AVE were observed.

### 2.4. Procedure

The first phase of the research was to validate the questionnaire designed specifically for this study, which originated in March 2019. Once the validity of the instrument was reached, the second phase consisted of intentional selection of the participants, through contact by the researchers with the physical education department of an educational center in the region previously described. The professionals of this department showed total interest in and collaboration with the study. The third phase was based on configuration of the analysis groups, which was random, as the school has two lines (A and B), resulting in group A, control, and group B, experimental. The fourth phase was based on the teaching of a didactic unit, in which group A followed a traditional methodology without the use of ICT resources and group B an innovative one by means of flipped learning, in its formative aspect of situational investment. This modality is based both on watching videos in the classroom and on the use of didactic software to improve the assimilation of content [21,22,23]. After the teaching unit, the last two phases of the study took place. Data collection was carried out in a room of the educational center, isolated from outside noise and with good lighting and ventilation, in order to ensure that the participants filled out the questionnaire in the best conditions. All the information obtained was treated following the ethical principles of research. Through a consent form, students were informed that their data would be treated in a manner that would preserve their anonymity, privacy, and confidentiality. Finally, all the information was exported to the statistical program for in-depth analysis.

## 3. Results

The results in Table 4 contain the scores obtained for the control groups (traditional methodology) during the application of the teaching unit in each educational stage. In general, the results obtained for the control group are very low. Of the nine variables analyzed, only one in primary education exceeded the central score (M ≥ 2.5) in the secondary stage. In primary education, no variable exceeded the central score. The variables with the highest score in the traditional methodology were the use of class time in primary education and the resolution of problems in secondary education. The interaction of students with the teacher and with classmates reached very low values.

The scores obtained for the experimental groups (Table 5) reflect the optimal effectiveness of inverted learning. Of the nine variables analyzed, the use of flipped learning allowed them to exceed the central score (M ≥ 2.5) for eight of the variables in primary and secondary education. Interaction with the teacher and with classmates are the most potent variables in both stages, in addition to autonomy in secondary education. In both educational stages, critical thinking is the variable with the lowest score, but it is very close to the central score (M = 2.4 and M = 2.48, respectively).

**Figure 1:**
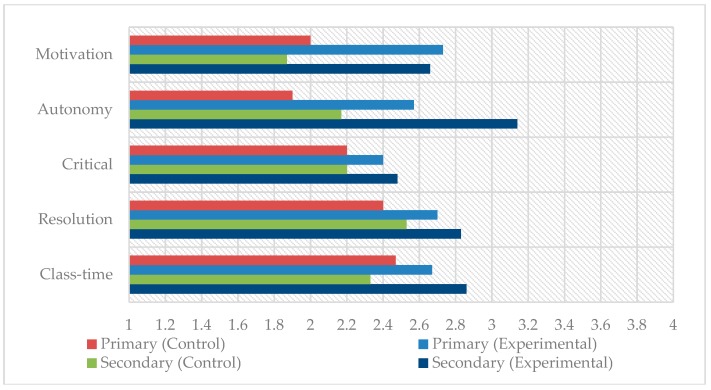
Intergroup comparison in attitudinal dimension.

In Figure 1, a comparison between the groups is made by means of a graph based on the means obtained in the attitudinal dimension. The means obtained for the students of the experimental group (with flipped learning methodology) are higher than those for the students of the control group (with traditional methodology), especially in the variables related to motivation and autonomy.

**Figure 2:**
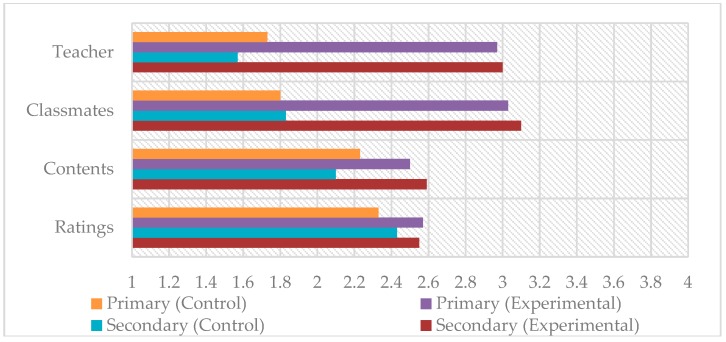
Intergroup comparison in interactive dimension.

On the other hand, Figure 2 shows a comparison of the means obtained for the experimental and control groups regarding the interactive dimension. Similar to the attitudinal dimension, the means obtained in the interactive dimension for the group with flipped learning is higher than those for the group with traditional methodology. The greatest difference was obtained with variables related to student interaction (with the teacher and with classmates).

To determine the value of independence between the results obtained for the traditional approach and flipped learning, Student’s t-test was carried out (Table 6). A standardized value of *p* < 0.05 was considered a statistically significant difference. As a corrective element for *d* (correlation force), a distinction for bi-serial correlation (*r* = [0, 1]) was made between small (*r* = −0.1), medium (*r* = −0.3), and large (*r* = −0.5) effect size.

It has been proven that the flipped learning approach in physical education causes a significant improvement in the interaction of students with teachers and peers. In both primary education (*r* = −0.57, *r* = −0.62) and secondary education (*r* = 0.63, *r* = −0.6), the results confirm an acceptable strength of association. Statistical significance was also obtained in the variables related to motivation and autonomy in both stages, obtaining a medium-low association in primary education (*r* = −0.35, *r* = −0.35) and secondary education (*r* = −0.41, *r* = −0.47), slightly higher for the latter. Finally, it highlights temporary use of the session (*r* = −0.28) and interaction with the didactic content (*r* = −0.27) in secondary education as significant variables, but with little association.

Finally, the results obtained for the experimental groups (in primary and secondary education) were analyzed to determine the value of the independence of flipped learning in physical education according to the educational stage (Table 7). Flipped learning was equally effective in both educational stages. A statistically significant difference was found only in the variable related to student autonomy (higher in secondary education). However, the analysis of effect size (*r* = −0.309) determined that this association in the final reflections should be viewed with caution (*r* < −0.5).

## 4. Discussion

The important role of technology in today’s society [1] should be to improve the training processes in different learning spaces generated in a digital era [2]. Educational technology must be used from a pedagogical perspective and exported to the different subjects that constitute educational curricula [79]. Thus, the novelty of this study consists in implementing the flipped learning method in the area of physical education, and also, as demonstrated by the beneficial results, developing the subject and acquiring the content by students. In addition, the results may be used to initiate a research path that uses educational stages in general and physical education in particular as incident factors in the use of flipped learning.

In this sense, ICT greatly facilitates the practice of teaching [3] and promotes the assimilation of meaningful and constructive learning by students [4]. In this line, the scientific literature reveals that techno-pedagogical means used in classrooms are beneficial for formative action [5]. In addition, the use of these digital resources enhances the improvement of a set of relevant academic indicators related to student performance and attitudes [6,7,8,9].

Therefore, the inclusion of ICT in different learning spaces at all educational stages should be promoted [10,11]. To put this into practice, the experiment presented in this work was developed. This study has allowed us to verify the effectiveness of flipped learning with respect to a traditional methodology in the field of physical education at two educational stages, primary and secondary education. This experiment provides continuity with previous reported works [53,54,55] verifying the potential of flipped learning as an innovative methodology. This experience also serves to confirm the innovative reform that physical education is undergoing today [56,57,58,59]. It should be noted that research analyzing the effects of flipped learning based on the educational stage is very scarce. No specific scientific studies have been reported in the field of education sciences that combine educational levels and physical education. This makes the discussion of results complex, and these cannot be compared directly.

After reviewing previous research, this study analyzed academic aspects such as motivation, autonomy, critical thinking, problem solving, the use of class time, interactions with teachers, peers, and other students, content, and the qualifications of evaluation tests. As has been proven in previous research, these study variables obtained positive evaluations after flipped learning was applied as an educational innovation, in contrast to the results achieved with students with whom a traditional teaching and learning methodology was used.

All academic indicators analyzed in this study obtained better scores for students who completed a training process through flipped learning. Specifically, the results of the present study are analogous with other precedents in variables such as motivation [27,28,30,31,47], autonomy [32], critical thinking, problem solving [36,37], the use of time in the classroom [19,20], teacher–student–content interactions [24,25,26,34,35,36], and academic performance [38,39].

Finally, this study shows that flipped learning is equally effective in teaching physical education in both primary and secondary education. Despite this and as mentioned above, these results cannot be debated since there are no specific studies in the scientific literature that analyze the use of flipped learning according to the educational stage of the students. Likewise, this demonstrates the effectiveness of this innovative approach, as stated in the literature [16,17,18]. Along these lines, its academic potential is verified against traditional teaching and learning methods [48,49,50].

## 5. Conclusions

In physical education, the use of flipped learning with students in primary education led to the improvement of all established indicators. Specifically, the most outstanding variables were the interactions of students with the teacher and with their classmates. In secondary education, similar results were found, encouraging the use of flipped learning to improve student interactions with teachers and peers, as well as empowerment of student autonomy in the learning process. On the other hand, the results obtained allow us to verify that the application of flipped learning did not enhance the development of critical thinking in any of the educational stages analyzed. Despite this, it is necessary to highlight that there were slight improvements in this variable with respect to the traditional methodology.

The comparative analysis between the traditional methodology and flipped learning allows us to conclude that flipped learning achieves greater potential in both educational variables, students’ autonomy, and interactions with the teacher and other students. Specifically, in addition to secondary education, the temporary nature of the session and the interaction of students with the content were improved.

With regard to the educational stage that achieved better results after the use of flipped learning, it was found that this approach is equally effective in primary and secondary education, highlighting autonomy exclusively as a significantly higher variable in secondary education.

Therefore, this research shows that flipped learning is an effective teaching and learning methodology in physical education. With flipped learning, better results were obtained in various academic indicators than with a traditional methodology that does not use technological resources to impart didactic content. Likewise, flipped learning showed great results in both educational stages, regardless of the level the students are enrolled in. It is concluded that the use of flipped learning in physical education is beneficial for students who are growing up in a digital age.

The expectation derived from the research focuses on the importance of promoting the inclusion of innovative methodologies in teaching and learning processes. In this case, the use of flipped learning brought great benefits in various established academic indicators. This situation demonstrates the potential of technology in today’s educational spaces.

The main limitation found in this study was in the digital competence of teachers to generate and impart content from an inverted perspective through the innovative methodology in question. Because the level of knowledge, skills, and digital skills of the teaching staff was not enough, the researchers had to actively collaborate with them. The researchers helped produce the audiovisual materials to deliver the sessions through flipped learning. In addition, the researchers had to recommend various applications, digital resources, and methodological guidelines to carry out flipped learning satisfactorily.

This study can lead to research on the use of flipped learning in physical education in other fields and with other content. The application of this study is totally practical, since it can be part of the daily and habitual reality of students and because it has practical results that can be measured and evaluated in terms of achieving objectives. Physical education specialists can discover a new way of acting in their classes, not only with the use of flipped learning, but also with other emerging methodologies, once their reliability and improvement in results have been proven, as in this case.

In addition to including innovation in the classroom, training and updating knowledge for professional development appropriate to the demands of a digital society should be encouraged. As a future line of study, we intend to analyze the digital competence of teachers who use flipped learning with the purpose of comparing methodological effectiveness with their level of digital skills.

## Figures and Tables

**Table 1:** Study groups by sex and educational stage.

**Boys**	**Primary Education**	**Secondary Education**
	***n* (%)**	***n* (%)**
Experimental group	16 (59.26)	11 (40.74)
Control group	12 (48)	13 (52)
Subtotal	28 (53.85)	24 (46.15)
**Girls**	**Primary Education**	**Secondary Education**
Experimental group	14 (43.75)	18 (56.25)
Control group	18 (51.43)	17 (48.57)
Subtotal	32 (47.76)	35 (52.24)

Source: own elaboration.

**Table 2:** Reliability and validity indices for the attitudinal factor.

Variables	α	CR	AVE	MSV	Motiva	Auton	Critical	Resolution	Class Time
Motivation	0.901	0.814	0.611	0.580	0.880				
Autonomy	0.905	0.908	0.601	0.470	0.511 *	0.719			
Critical	0.873	0.815	0.582	0.134	0.341 *	0.251 *	0.792		
Resolution	0.890	0.901	0.712	0.529	0.747 *	0.610 *	0.264 *	0.791	
Class time	0.901	0.921	0.604	0.417	0.611 *	0.582 *	0.271 *	0.703 *	0.713

* Significant correlation *p* < 0.001. Source: own elaboration. CR: Composite Reliability, AVE: Average Variance Extracted, MSV: Maximum shared squared variance.

**Table 3:** Reliability and validity indices for the interactive factor.

Factor	α	CR	AVE	MSV	Teacher	Classmates	Content
Teacher	0.903	0.815	0.613	0.215	0.899		
Classmates	0.901	0.812	0.807	0.631	0.384 *	0.898	
Content	0.899	0.843	0.625	0.510	0.311	0.804 *	0.766

* Significant correlation *p* < 0.001. Source: own elaboration.

**Table 4:** Results obtained for study variables in control group.

Variables		Likert Scale, *n* (%)				Parameters			
		None	Few	Enough	Completely	M	SD	Skew	Kurt
**Primary Education**	Motivation	10 (33.3)	12 (40)	6 (20)	2 (6.7)	2	0.89	1.19	−0.5
	Autonomy	12 (40)	11 (36.7)	5 (16.7)	2 (6.7)	1.9	0.91	0.99	−0.34
	Critical	7 (23.3)	13 (43.3)	7 (23.3)	3 (10)	2.2	0.91	1.32	−0.61
	Resolution	6 (20)	10 (33.3)	10 (33.3)	4 (13.3)	2.4	0.95	1.40	−0.94
	Class time	5 (16.7)	9 (30)	13 (43.3)	3 (10)	2.47	0.88	1.66	−0.76
	Teacher	15 (50)	10 (33.3)	3 (10)	2 (6.7)	1.73	0.89	0.82	0.42
	Classmates	10 (33.3)	16 (53.3)	4 (13.3)	0 (0)	1.8	0.65	1.22	−0.73
	Content	6 (20)	13 (43.3)	9 (30)	2 (6.7)	2.23	0.84	1.46	−0.6
	Ratings *	4 (13.3)	14 (46.7)	10 (33.3)	2 (6.7)	2.33	0.79	1.69	−0.4
**Secondary Education**	Motivation	11 (36.7)	14 (46.7)	3 (10)	2 (6.7)	1.87	0.85	1.02	0.42
	Autonomy	7 (23.3)	14 (46.7)	6 (20)	3 (10)	2.17	0.9	1.3	−0.44
	Critical	7 (23.3)	12 (40)	9 (30)	2 (6.7)	2.2	0.87	1.38	−0.74
	Resolution	5 (16.7)	9 (30)	11 (36.7)	5 (16.7)	2.53	0.96	1.6	−0.93
	Class time	5 (16.7)	13 (43.3)	9 (30)	3 (10)	2.33	0.87	1.53	−0.61
	Teacher	17 (56.7)	10 (33.3)	2 (6.7)	1 (3.3)	1.57	0.76	0.74	1.54
	Classmates	11 (36.7)	13 (43.3)	6 (20)	0 (0)	1.83	0.73	1.13	−1.11
	Content	7 (23.3)	15 (50)	6 (20)	2 (6.7)	2.1	0.83	1.32	−0.18
	Ratings *	3 (10)	13 (43.4)	12 (40)	2 (6.7)	2.43	0.76	1.88	−0.37

* Sample grouping of ratings (min: 0; max: 10) was carried out based on the following criteria: none: 0–4.9; few: 5–5.9; enough: 6–8.9; completely: 9–10. Source: own elaboration.

**Table 5:** Results obtained for study variables in experimental group.

Variables		Likert Scale, *n* (%)				Parameters			
		None	Few	Enough	Completely	M	SD	Skew	Kurt
**Primary Education**	Motivation	5 (16.7)	6 (20)	11 (36.7)	8 (26.7)	2.73	1.03	1.68	−1.01
	Autonomy	3 (10)	12 (40)	10 (33.3)	5 (16.7)	2.57	0.88	1.78	−0.76
	Critical	5 (16.7)	11 (36.7)	11 (36.7)	3 (10)	2.4	0.88	1.59	−0.74
	Resolution	4 (13.3)	6 (20)	15 (50)	5 (16.7)	2.7	0.9	1.89	−0.5
	Class time	4 (13.3)	8 (26.7)	12 (40)	6 (20)	2.67	0.94	1.77	−0.83
	Teacher	3 (10)	4 (13.3)	14 (46.7)	9 (30)	2.97	0.91	2.16	0.18
	Classmates	2 (6.7)	5 (16.7)	13 (43.3)	10 (33.3)	3.03	0.87	2.32	−0.23
	Content	4 (13.3)	9 (30)	15 (50)	2 (6.7)	2.5	0.81	1.86	−0.49
	Ratings *	3 (10)	11 (36.7)	12 (40)	4 (13.3)	2.57	0.84	1.86	−0.6
**Secondary Education**	Motivation	4 (13.8)	7 (24.1)	13 (44.8)	5 (17.2)	2.66	0.92	1.8	−0.7
	Autonomy	2 (6.9)	4 (13.8)	11 (37.9)	12 (41.1)	3.14	0.9	2.38	−0.09
	Critical	5 (17.2)	9 (31)	11 (37.9)	4 (13.8)	2.48	0.93	1.59	−0.88
	Resolution	4 (13.8)	6 (20.7)	10 (34.5)	9 (31)	2.83	1.02	1.79	−0.94
	Class time	3 (10.3)	6 (20.7)	12 (41.4)	8 (27.6)	2.86	0.94	1.99	−0.64
	Teacher	3 (10.3)	5 (17.2)	10 (34.5)	11 (37.9)	3	0.98	2.04	−0.63
	Classmates	2 (6.9)	4 (13.8)	12 (41.1)	11 (37.9)	3.1	0.88	2.38	−0.06
	Content	4 (13.8)	8 (27.6)	13 (44.8)	4 (13.8)	2.59	0.89	1.78	−0.67
	Ratings *	4 (13.8)	9 (31)	12 (41.4)	4 (13.8)	2.55	0.89	1.74	−0.73

* Sample grouping of ratings (min: 0; max: 10) was carried out based on the following criteria: none: 0–4.9; few: 5–5.9; enough: 6–8.9; completely: 9–10. Source: own elaboration.

**Table 6:** Study of the value of independence between control and experimental groups.

Variables		Group, M (SD)		M_2_−M_1_	Student’s t		*d*	*r*
		Control	Experimental		*t* (df)	*p*-Value		
**Primary education**	Motivation	2 (0.89)	2.73 (1.03)	0.73	2.89 (58)	0.005	−0.758	−0.354
	Autonomy	1.9 (0.91)	2.57 (0.88)	0.67	2.55 (58)	0.014	−0.748	−0.351
	Critical	2.2 (0.91)	2.4 (0.88)	0.2	0.85 (58)	0.398	-	-
	Resolution	2.4 (0.95)	2.7 (0.9)	0.3	1.23 (58)	0.223	-	-
	Class time	2.47 (0.88)	2.67 (0.94)	0.2	0.83 (58)	0.408	-	-
	Teacher	1.73 (0.89)	2.97 (0.91)	1.24	5.21 (58)	<0.001	−1.378	−0.567
	Classmates	1.8 (0.65)	3.03 (0.87)	1.23	6.08 (58)	<0.001	−1.602	−0.625
	Content	2.23 (0.84)	2.5 (0.81)	0.32	1.23 (58)	0.224	-	-
	Ratings	2.33 (0.79)	2.57 (0.84)	0.24	1.09 (58)	0.281	-	-
**Secondary education**	Motivation	1.87 (0.85)	2.66 (0.92)	0.79	3.36 (57)	0.001	−0.892	−0.407
	Autonomy	2.17 (0.9)	3.14 (0.9)	0.97	4.08 (57)	<0.001	−1.078	−0.474
	Critical	2.2 (0.87)	2.48 (0.93)	0.28	1.18 (57)	0.242	-	-
	Resolution	2.53 (0.96)	2.83 (1.02)	0.3	1.12 (57)	0.266	-	-
	Class time	2.33 (0.87)	2.86 (0.94)	0.53	2.21 (57)	0.031	−0.585	−0.281
	Teacher	1.57 (0.76)	3 (0.98)	1.43	6.14 (57)	<0.001	−1.631	−0.632
	Classmates	1.83 (0.73)	3.1 (0.88)	1.27	5.89 (57)	<0.001	−1.571	−0.618
	Content	2.1 (0.83)	2.59 (0.89)	0.49	2.13 (57)	0.038	−0.569	−0.274
	Ratings	2.43 (0.76)	2.55 (0.89)	0.12	0.54 (57)	0.593	-	-

Source: own elaboration. M_1_: Mean of the experimental group, M_2_: Mean of the control group.

**Table 7:** Study of the value of independence between experimental groups.

Variables	Group, M (SD)		M_2_−M_1_	Student’s *t*		*D*	*r*
	Primary	Secondary		*t* (df)	*p*-Value		
Motivation	2.73 (1.03)	2.66 (0.92)	−0.07	0.3 (57)	0.764	-	-
Autonomy	2.57 (0.88)	3.14 (0.9)	0.57	2.14 (57)	0.019	−0.64	−0.305
Critical	2.4 (0.88)	2.48 (0.93)	0.08	0.34 (57)	0.732	-	-
Resolution	2.7 (0.9)	2.83 (1.02)	0.13	0.5 (57)	0.619	-	-
Class time	2.67 (0.94)	2.86 (0.94)	0.19	0.78 (57)	0.436	-	-
Teacher	2.97 (0.91)	3 (0.98)	0.03	0.13 (57)	0.895	-	-
Classmates	3.03 (0.87)	3.1 (0.88)	0.07	0.3 (57)	0.765	-	-
Content	2.5 (0.81)	2.59 (0.89)	0.09	0.53 (57)	0.598	-	-
Ratings	2.57 (0.84)	2.55 (0.89)	−0.02	0.06 (57)	0.949	−	−

Source: own elaboration. M_1_: Mean of the Secondary group, M_2_: Mean of the Primary group.
